# Progress and developments towards making historic administrative data research ready

**DOI:** 10.23889/ijpds.v9i5.2891

**Published:** 2024-09-18

**Authors:** Lee Williamson, Chris Dibben

**Affiliations:** 1 University of Edinburgh

## Objectives

To make research ready data from transcribed historic civil registration records: birth, marriage and death (from mid-19th century onwards). To use historic records effectively for large-scale research they must not only be made machine-readable, but also coded in a suitable format – along with classification of transcribed information. Included on the digitised historic records are textual descriptions of occupations and causes of death. Thus, to code transcribed occupations to HISCO and causes of death to ICD.

## Approach

It is impractical to hand-code the records manually (34 million occupations and 8 million causes of death), especially for deaths where more than one cause can be given. As such, coding is viewed as a text classification task and the process is automated. To facilitate auto-coding, a proportion of records were hand-coded as training data (90,000 occupations and 102,000 deaths). Ahead of the auto-coding, initial pre-processing, cleaning and standardising is done on both the occupations and deaths.

## Results

Preliminary experiments undertaken obtained reasonable results from a combination of exact matching and statistical classification. Experiments using a larger pilot uncovered that since some occupations are very common, the training data set covers a very large proportion of the records (ie exact match). This proportion is not as high for deaths given the different ways causes are written.

## Conclusion

This is work in progress to create the research ready data, and the poster will include the results from experiments using the full training data (90,000 occupations and 102,000 deaths).

